# The Prognostic Value of Family History for the Estimation of Cardiovascular Mortality Risk in Men: Results from a Long-Term Cohort Study in Lithuania

**DOI:** 10.1371/journal.pone.0143839

**Published:** 2015-12-02

**Authors:** Abdonas Tamosiunas, Ricardas Radisauskas, Jurate Klumbiene, Gailute Bernotiene, Janina Petkeviciene, Dalia Luksiene, Dalia Virviciute, Vilija Malinauskiene, Olga Vikhireva, Vilius Grabauskas

**Affiliations:** 1 Institute of Cardiology, Academy of Medicine, Lithuanian University of Health Sciences, Kaunas, Lithuania; 2 Faculty of Public Health, Academy of Medicine, Lithuanian University of Health Sciences, Kaunas, Lithuania; 3 Department of Epidemiology and Public Health, University College London, London, United Kingdom; Weill Cornell Medical College Qatar, QATAR

## Abstract

**Aim:**

To evaluate the additional prognostic value of family history for the estimation of cardiovascular (CVD) mortality risk in middle-aged urban Lithuanian men.

**Methods:**

The association between family history of CVD and the risk of CVD mortality was examined in a population-based cohort of 6,098 men enrolled during 1972–1974 and 1976–1980 in Kaunas, Lithuania. After up to 40 years of follow-up, 2,272 deaths from CVD and 1,482 deaths from coronary heart disease (CHD) were identified. Multivariate Cox proportional hazards models were used to estimate hazard ratios (HR) for CVD and CHD mortality.

**Results:**

After adjustment for traditional CVD risk factors, the HR for CVD mortality was 1.24 (95% CI 1.09–1.42) and for CHD mortality 1.20 (1.02–1.42) in men with first-degree relatives having a history of myocardial infarction (MI), compared to men without positive family history. A significant effect on the risk of CVD and CHD mortality was also observed for the family history of sudden cardiac death and any CVD. Addition of family history of MI, sudden death, and any CVD to traditional CVD risk factors demonstrated modest improvement in the performance of Cox models for CVD and CHD mortality.

**Conclusions:**

Family history of CVD is associated with a risk of CVD and CHD mortality significantly and independently of other risk factors in a middle-aged male population. Addition of family history to traditional CVD risk factors improves the prediction of CVD mortality and could be used for identification of high-risk individuals.

## Introduction

Several prospective and case-control studies have shown that family history of CHD, stroke, and diabetes is a risk factor of cardiovascular disease (CVD) independent of well-established risk factors, such as age, sex, smoking, arterial hypertension (AH), obesity, and dyslipidemia [1–3]. Family history reflects the complex interactions of genetic and non-genetic risk factors, for example, behaviours and cultural factors, and may influence a person’s perception of CVD risk and participation in the preventive actions [4, 5].

Recent prospective studies have demonstrated that cardiovascular risk prediction could be improved by adding family history of CVD to conventional risk factors in the prognostic models [6, 7]. Therefore, family history has been shown to be a potential screening tool to identify subjects at increased risk who may be candidates for enhanced prevention strategies [8].

High mortality from CVD is a major health problem in the Lithuanian male population. During the last decades, the increasing trend of CVD mortality was observed in Lithuanian men aged 45–59 years, reaching 482.5 per 100 000 population in 2012 and being one of the highest in Europe [9]. Epidemiological studies demonstrated that the prevalence of conventional CVD risk factors is also very high in Lithuanian population [10]. Prognostic values of these risk factors for the development of CVD in Lithuania have been found to be comparable to other populations [11, 12]. However, the impact of family history on the prediction of mortality from CVD and other diseases has never been evaluated in Lithuania and other countries of Central and Eastern Europe.

The aim of this study was to assess the additional prognostic value of family history for the estimation of CVD mortality risk in middle-aged urban Lithuanian men.

## Materials and Methods

### Study design and sample

The study used the data from two population-based cohorts participating in Kaunas Rotterdam Intervention Study (KRIS) and Multifactorial Ischemic Heart Disease Prevention Study (MIHDPS). In KRIS, a random sample of men aged 45–59 years, living in the city of Kaunas (Lithuania), was examined during the years 1972–1974. In MIHDPS, a random sample of Kaunas men aged 40–59 years was examined between 1976 and 1980. In total, 8,380 men participated in baseline surveys—2,447 men in KRIS (participation rate 69.2%) and 5,933 men in MIHDPS (participation rate 69.8%). We excluded 242 (2.9%) men because of incomplete information on family history of CVD and 1264 (15.1%) men who lacked any information about the CVD risk factors included in Cox models. Excluded persons did not differ from the rest of the cohort according to other analysed variables. In addition, 776 (9.3%) men with CVD at baseline were excluded from the analysis of CVD mortality risk. The final number of participants included in the present analysis was 6,098.

Both studies were based on voluntary, informed participation. Participants did not provide written consent prior to the baseline examination, as this was not required in the former Soviet Union. The study protocol was approved by the Regional Biomedical Research Ethics Committee at Lithuanian University of Health Sciences. Participants’ records and information were anonymized and de-identified prior to analysis.

### Measurements

The questionnaires used in both studies were very similar. In each survey, CVD risk factors were defined using the same methodology.

BP was measured on the right brachial artery using a mercury sphygmomanometer and appropriately sized arm cuffs in the sitting position after 5 minutes of rest. The measurements were performed to the nearest 2 mmHg. The first Korotkoff phase was recorded as systolic BP, and the fifth Korotkoff phase was used to determine diastolic BP. The average of two measurements was used in the analysis. AH was defined as systolic BP of at least 140 mm Hg and/or diastolic BP of at least 90 mm Hg, and/or antihypertensive medication for the last two weeks before examination. The height of participants was measured to the nearest centimetre with a stadiometer. Weight was measured without shoes or heavy clothes to the nearest 0.1 kg with standardized medical scales. Body mass index (BMI) was calculated as weight in kilograms divided by height in meters squared (kg/m^2^). Overweight was defined as BMI 25.0–29.99 kg/m^2^, and obesity as BMI ≥30.0 kg/m^2^.

For both studies, fasting serum samples were analyzed in the Laboratory of the Institute of Cardiology, Lithuanian University of Health Sciences. Serum total cholesterol concentration was measured by the method of Huang et al. [13]. Fasting glucose concentration was directly determined in serum by the ortho-toluidine method [14].

A standard questionnaire was applied to obtain data on the respondent’s age, physical activity, smoking status, alcohol consumption, antihypertensive, lipid-lowering, or antidiabetic medications taken, and family history of CVD. Physical activity was assessed by hours spent on leisure-time moderate activity per week. The respondents were categorized into two groups according to their level of physical activity: active (≥10 hours/week) and inactive (<10 hours/week). According to alcohol consumption levels, the respondents were classified into six groups: never or former drinkers, those consuming alcoholic beverages less frequently than once per month, 1–3 times per month, once per week, 2–3 times per week, several times per week, or daily. Smoking status of participants was categorized as never smokers, ex-smokers, and daily smokers.

CHD at baseline was determined by: 1) a documented history of MI and/or ischemic changes on electrocardiogram (ECG) coded by Minnesota codes (MC) 1–1 or 1–2 [15]; 2) angina pectoris as defined by G. Rose’s questionnaire (without MI and/or MC 1–1 or 1–2) [16]; 3) ECG findings coded by MC 1–3, 4–1, 4–2, 4–3, 5–1, 5–2, 5–3, 6–1, 6–2, 7–1, or 8–3 (without MI and/or MC 1–1, 1–2 and without angina pectoris). Previous stroke was determined according to a documented history of stroke.

The information on family history of MI, stroke, and sudden death was evaluated among first-degree relatives only: parents (father and mother) and siblings (brothers and/or sisters). The following questions were asked: “Did your father ever experience MI (stroke or sudden death)?”, “Did your mother ever experience MI (stroke or sudden death)?”, “Did your brother ever experience MI (stroke or sudden death)?”, and “Did your sister ever experience MI (stroke or sudden death)?”. We also used a combined family history variable—family history of CVD—which included history of MI and/or stroke and/or sudden death among at least one of the parents or siblings. According to the family history variable, the participants were categorized as having one or more parents or siblings with CVD and not having any first-degree relatives with CVD.

### Follow-up

The data from the regional mortality register based on death certificates were used for the follow-up of participants. Only underlying causes of death were considered. Death certificates based on medical documentation were sufficiently valid and complete [17]. Deaths between the baseline survey date and December 31^st^ 2013 were registered. Causes of death were coded by the International Classification of Diseases (ICD) (versions 9 and 10): deaths of CVD included codes 390–458 of ICD-9 and codes I00-I99 of ICD-10; deaths from CHD included codes 410–414 of ICD-9 and codes I20-I25 of ICD-10. Over the period of 1972–2013, 4,574 death cases from any cause, 2,272 deaths from CVD, and 1,482 deaths from CHD were registered. The mean duration of follow-up was 24.1±10.3 years.

### Statistical analysis

Descriptive statistics were calculated for variables included in the data analysis. We calculated the Systematic Coronary Risk Evaluation (SCORE) values using the high-risk version of SCORE, recommended by the European Society of Cardiology [18].

The Kaplan-Meier survival curves for cumulative CVD and CHD mortality by the number of first-degree relatives with CVD were plotted, and the log-rank tests for the difference in cumulative mortality were applied. Hazard ratios (HR) and 95% confidence intervals (CI) were estimated by the multivariate Cox proportional hazards regression for CVD and CHD mortality separately. Attained age in months was used as time-scale. Several models were assessed. Model 1 included the SCORE variable and other risk factors: alcohol intake frequency, physical activity, BMI, fasting glucose level, and use of antihypertensive, antidiabetic, or lipid-lowering drugs. Other four models included variables of Model 1 plus family history of MI (Model 2), or family history of stroke (Model 3), or family history of sudden death (Model 4), or family history of any CVD (Model 5).

We compared the predictive ability of Cox regression models extended by family history with the predictive ability of Model 1. The additional predictive value of the models extended by family history was determined using likelihood ratio test (LRT). The ability of the models to predict CVD and CHD mortality was also estimated calculating the Harrell’s C statistic (an established measure of model discrimination for binary outcomes), and integrated discrimination improvement (IDI; a relatively independent of risk thresholds and categories measure of the ability of the extended model to improve average sensitivity without compromising average specificity) [19, 20].

Data were analyzed using the IBM SPSS Statistics 20.0 and Stata/IC 10.0. P<0.05 values were considered statistically significant.

## Results

The distribution of men by the presence of first-degree relatives with a history of MI, stroke, sudden death, and any CVD in four age groups is presented in Table 1. The prevalence of positive family history of MI, stroke, sudden death, and any CVD was 9.2%, 9.3%, 11.4%, and 23.5%, respectively. The proportion of subjects who had first-degree relatives with stroke, sudden death, or any CVD increased with age at baseline.

**Table 1:** Distribution of men by the family history of CVD in age groups.

Family history of CVD		Age	group,	years		p
	40–44	45–49	50–54	55–59	Total	
	N = 1150	N = 2003	N = 1677	N = 1268	N = 6098	
	%	%	%	%	%	
**MI**						
No	90.5	90.1	91.3	91.7	91.7	0.391
Yes	9.5	9.9	8.7	8.3	9.2	
**Stroke**						
No	93.5	91.5	89.5	88.4	90.6	<0.001
Yes	6.5	8.5	10.5	11.6	9.3	
**Sudden death**						
No	90.8	89.8	86.9	86.9	88.6	0.001
Yes	9.2	10.2	13.1	13.1	11.4	
CVD*						
No	79.9	76.7	74.8	75.2	76.5	0.009
Yes	20.1	23.3	25.2	24.8	23.5	

Abbreviations: CVD—cardiovascular disease, MI—myocardial infarction.

CVD*—history of MI or/and stroke or/and sudden death.

The mean values of risk factors and SCORE, as well as the prevalence of risk factors, among men according to the presence of first-degree relatives with history of CVD and age at baseline are presented in S1 Table. Men with positive family history of CVD had higher levels of total cholesterol (age group 50–54 years), BMI, and fasting glucose (age group 55–59 years), compared to men without family history of CVD. Having first-degree relatives with CVD was related to a lower prevalence of low physical activity in the age group 45–49 years and a higher prevalence of frequent consumption of alcohol (one or more times per week) in the age group 50–54 years.

**Fig 1:**
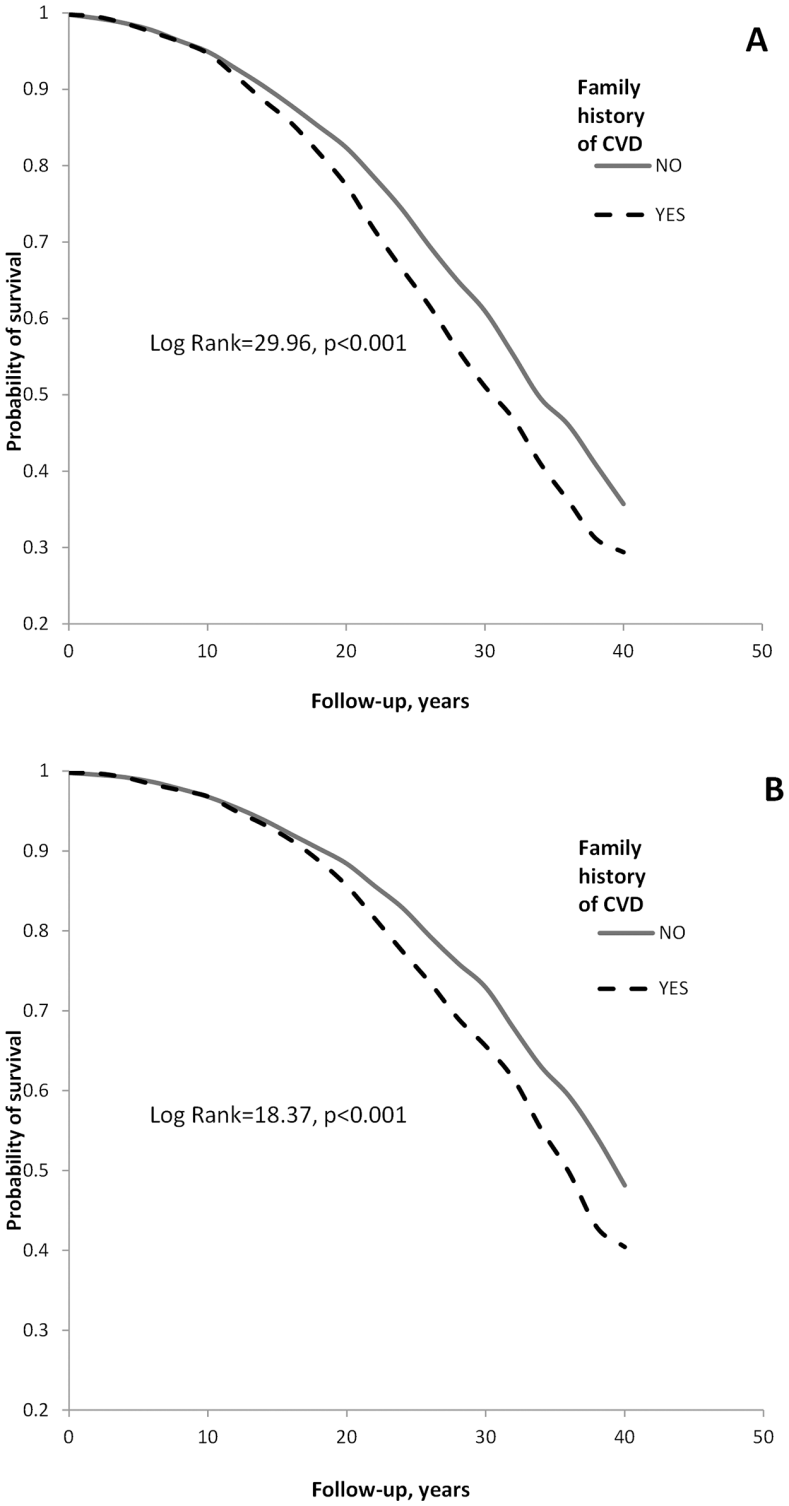
Probability of survival for cardiovascular disease (CVD) mortality (panel A) and coronary heart disease (CHD) mortality (panel B) according to the presence of first-degree relatives with CVD (myocardial infarction or/and stroke or/and sudden death) .

Fig 1 shows the Kaplan-Meier survival curves for cumulative CVD and CHD mortality among men having and not having first-degree relatives with CVD. A clear pattern of lower survival rates (log-rank p values <0.001) was observed among men with positive family history of CVD.

After multivariate adjustment for SCORE, alcohol intake frequency, physical activity, BMI, fasting glucose level, and use of antihypertensive, antidiabetic, or lipid-lowering medications, men with a family history of MI had a 24% higher risk of fatal CVD mortality and a 20% higher risk of CHD mortality, as compared to men who had no family history of MI (Tables 2 and 3).

**Table 2:** Comparison of models for prediction of CVD mortality without and with addition of CVD family history.

Firstdegreerelativeswith CVD			Models		
	Model 1	Model 2	Model 3	Model 4	Model 5
		(FH of MI)	(FH of stroke)	(FH of sudden death)	(FH of CVD)
	HR	HR	HR	HR	HR
	(95% CI)	(95% CI)	(95% CI)	(95% CI)	(95% CI)
No	-	1	1	1	1
Yes		1.24	1.09	1.20	1.23
		(1.09–1.42)	(0.95–1.24)	(1.07–1.36)	(1.12–1.34)
Harrell‘s C	0.6807	0.6810	0.6809	0.6817	0.6825
LRT, p		0.002	0.237	0.004	<0.001
IDI, %,		1.25	0.19	1.93	2.32
p		<0.001	<0.001	<0.001	<0.001

Model 1—SCORE and factors not included into SCORE: alcohol intake frequency, physical activity, body mass index, fasting glucose level, and use of antihypertensive, antidiabetic, or lipid-lowering medications; Model 2 –variables of Model 1 plus family history of MI; Model 3—variables of Model 1 plus family history of stroke; Model 4—variables of Model 1 plus family history of sudden death; Model 5—variables of Model 1 plus family history of MI and/or stroke and/or sudden death.

Abbreviations: CI—confidence interval, CVD—cardiovascular disease, FH—family history, HR—hazard ratios, IDI—integrated discrimination improvement, LRT—likelihood ratio test, MI—myocardial infarction, SCORE—Systematic Coronary Risk Evaluation.

**Table 3:** Comparison of models for prediction of CHD mortality without and with addition of CVD family history.

First-degreerelativeswith CVD			Models		
	Model 1	Model 2	Model 3	Model 4	Model 5
		(FH of MI)	(FH of stroke)	(FH of sudden death)	(FH of CVD)
	HR	HR	HR	HR	HR
	(95% CI)	(95% CI)	(95% CI)	(95% CI)	(95% CI)
No	-	1	1	1	1
Yes		1.20	1.02	1.21	1.22
	-	(1.02–1.42)	(0.86–1.21)	(1.04–1.41)	(1.09–1.37)
Harrell‘s C	0.6793	0.6791	0.6789	0.6803	0.6806
LRT, p	-	0.035	0.833	0.014	0.001
IDI, %,	-	1.20	0.0	2.30	2.50
p	-	<0.05	>0.05	<0.01	<0.05

Model 1—SCORE and factors not included into SCORE: alcohol intake frequency, physical activity, body mass index, fasting glucose level, and use of antihypertensive, antidiabetic, or lipid-lowering medications; Model 2 –variables of Model 1 plus family history of MI; Model 3—variables of Model 1 plus family history of stroke; Model 4—variables of Model 1 plus family history of sudden death; Model 5—variables of Model 1 plus family history of MI and/or stroke and/or sudden death.

Abbreviations: CHD—coronary heart disease, CI—confidence interval, CVD—cardiovascular disease, FH—family history, HR—hazard ratios, IDI—integrated discrimination improvement, LRT—likelihood ratio test, MI—myocardial infarction, SCORE—Systematic Coronary Risk Evaluation.

The risk of CVD and CHD mortality also increased for men having first-degree relatives with family history of sudden death and any CVD. The family history of stroke was not significantly related to either CVD or CHD mortality risk.

The comparison of Cox regression models showed that the addition of family history had some effect on models’ performance. According to the LRT, adding family history of MI, sudden death, and CVD demonstrated improved performance of the Cox models for both CVD and CHD mortality. A slight increase in Harrell’s C-statistic was observed, once family history of MI, stroke, sudden death or combined family history of CVD were added to the SCORE variable and other risk factors in the models for CVD mortality. In all models, the values of IDI were 0.19–2.32% for CVD mortality and 0.20–2.30% for CHD mortality, which suggests a modest improvement in discrimination of the Cox regression models when family history variables were added.

## Discussion

In this long-term prospective study, we analysed the prevalence and predictive value of family history of CVD for mortality from CVD and CHD among middle-aged men. To our knowledge, our study is the first in Baltic countries to show a positive association between family history of CVD and mortality from CVD and CHD in a large cohort sample of men.

Our definition of positive family history included the history of MI, stroke, and sudden death among first-degree relatives only: among parents (father and/or mother) and among siblings (brothers and/or sisters). We also used the combined family history—family history of any CVD (either MI, or stroke, or sudden death, or their combination among first-degree relatives).

Although the term “family history of CVD (or CHD)” is frequently used, there is no common or uniform definition at the moment. Most definitions include statements of either “family history of CVD” or “family history of CVD (or CHD, or stroke, or heart attack) among first-degree relatives” and are treated as a binary variable [21, 22]. The current most common definition of family history of CVD also includes the age of CVD occurrence: early (premature) CVD (at age before 55 or 60 years for men and before 65 years for women) and late CVD in first-degree relatives [23, 24]. Other characteristics taken into account by the family history definition are the degree of relationship (first-degree (parents and siblings) or second-degree (grandparents) relatives), type of relative (parent, sibling, child, or grandparent), number of relatives with a positive history, and lineage (maternal or paternal) [6, 25, 26].

In other countries, the results of cross-sectional and prospective studies show that from 6.5% to 50% of respondents have a first or second-degree relative with CVD [7, 8, 22, 27, 28]. The differences in the prevalence of family history of CVD found in epidemiological studies might be explained by the differences in study samples (age, gender, disease status (free from CVD or with CVD at baseline)) and different definitions of the family history of CVD. Some studies used a very strict definition of the family history of CVD, when only one specific form of the disease (for example, MI, or heart attack, or stroke) was included in the definition of positive family history [8, 23, 29, 30]. In contrast to these studies, other investigators used a very broad definition of the family history of CVD. In a prospective cohort study of 6,435 British men aged 35–55 years, the participants were asked whether either of their parents had a stroke, a heart attack, or angina. Family history of CHD was considered positive if either or both parents suffered from any of these diseases [31]. An even broader definition of the family history of CVD was used by a longitudinal population study in a random sample of men and women aged 35–55 years from Erpe-Mere and Nieuwerkerken in Belgium. The definition of a positive family history included both fatal and non-fatal CVD, such as MI, coronary revascularisation, peripheral vascular intervention on inguinal or lower limb arteries, stroke, carotid revascularisation, or sudden cardiac death [32]. Other studies used the definition of family history of CVD or CHD without specifying what forms of the disease were considered [22, 24]. In our study, we asked participants about specific forms of both fatal and non-fatal CVD among their parents and siblings: MI, stroke, and sudden death. This approach also enabled us to use a broader definition of positive family history—family history of any CVD.

In our cohorts, family history of CVD was associated with higher levels of some CVD risk factors as compared to men without family history. Other studies also found that family history of CVD was related to higher levels of main CVD risk factors [1, 8]. On the other hand, the multinational case-control INTERHEART study data showed that individuals with family history of MI had lower levels of behavioural CVD risk factors [33]. It was suggested that the respondents with a positive family history might have already changed their health behaviours due to a greater awareness of the impact of unhealthy lifestyle on CVD risk. The higher levels of biological risk factors in persons having family history of CVD indicate the need for more intensive CVD prevention [34].

In our study, respondents with a family history of MI, sudden death, and CVD among first-degree relatives had a higher risk of CVD and CHD mortality, after adjustment for SCORE, BMI, fasting glucose, alcohol intake frequency, physical activity, and use of antihypertensive, antidiabetic, or lipid-lowering medications. Adding family history of MI, sudden death, and any CVD improved the predictive performance of Cox regression models for CVD and CHD mortality. Our findings are consistent with other studies reporting an association between parental CVD and the offspring incidence and mortality from CHD or CVD [22, 24, 28]. Similar to our results, other studies also demonstrated moderate to modest improvement in the prognostic performance of CVD and CHD risk models extended by family history of CVD [1, 6, 7, 32]. The increase in C-statistic was, for example, from 0.718 to 0.725 in male participants of the Multi-Ethnic Study of Atherosclerosis [6], and from 0.699 to 0.708 among men participating in the Physicians’ Health Study II [7]. Net reclassification improvement, which, like IDI, is a summary measure of the model performance improvement, ranged from 2% to 5% [1, 6, 7]. The relatively small effect of family history on CVD risk prediction could be largely explained by the mediation of this effect via conventional risk factors [1].

The strengths of our study are as follows. First, it was a long-term, large-scale cohort-based study among urban men, with over 2,200 deaths registered during more than 40 years of the follow-up. Second, multiple lifestyle and biological risk factors of CVD and other variables were assessed in the baseline survey. Third, adjustments were made for a large number of potential confounding variables in the Cox models. The mentioned advantages provided adequate statistical power to assess the impact of family history of CVD among parents and siblings on the risk of CVD and CHD mortality in the sample of middle-aged urban men.

Several limitations of our study should be acknowledged. First, we did not have information on the age of CVD onset among parents and siblings. Therefore, we were unable to investigate the association between parental history of the early-onset CVD and fatal CVD in offspring. For early-onset CVD one would expect the association of interest be stronger than for late-onset CVD, due to a predominant effect of genetic factors for the former and conventional risk factors for the latter [1, 6, 28, 32; 35, 36]. Combining early-onset and late-onset CVD into one exposure variable could, therefore, dilute the association between positive family history of CVD and the risk of fatal CVD. Second, family history of CVD was self-reported and not validated. In the studies which estimated the accuracy of self-reported family history of CVD in first-degree relatives, sensitivity ranged between 50% and 90%, whereas specificity in most cases was higher than 80% [37, 38]. Considering that our study participants were asked about CVD in their first-degree relatives, recall bias was more likely to manifest in over-reporting, rather than under-reporting, of family history of myocardial infarction, stroke, or sudden death. Such potential over-reporting could increase the number of “false exposed” and reduce the strength of our association of interest. Third, we reported family history for men aged 40–59 years. In the selected age group of respondents, some men could have parents (particularly mothers) who were too young to have experienced MI or stroke. Unfortunately, we did not perform follow-up surveys of the cohorts and do not have data on changes of risk factors and family history. The baseline levels of cardiovascular risk factors were likely to change during the follow-up period (for example, due to the start of antihypertensive, lipid-lowering, or antidiabetic pharmacotherapy). This could result in potential regression dilution bias, or underestimation of the association of interest. Similarly, the change in family history during follow-up (inability to take into account the post-baseline CVD events in first-degree relatives and inflation of the number of “false non-exposed”) could weaken this association. However, the general concept of risk prediction implies the estimation of the future outcome risk based on the current exposure levels. While the above-mentioned limitations could affect our results, we believe that the association of interest was not inflated, and its genuine strength is at least similar to or greater than our estimates. Finally, the present study focused on a random male population sample in one urban setting, rather than on a nationally representative sample of men and women. Women were not screened in the baseline surveys, because in the 1980s, CVD mortality was extremely high in Lithuanian men, and epidemiological studies were focused on the middle-aged male population. While our sample was not nationally representative, the CVD mortality rates were comparable with the national data [9]).

## Conclusions

Family history of CVD among middle-aged men is associated with CVD and CHD mortality significantly and independently of other well-known lifestyle and biological risk factors. The strongest associations were observed in persons who had first-degree relatives with a history of MI and sudden cardiac death. Our findings provide further evidence that addition of family history to traditional CVD risk factors slightly improves the prediction of CVD mortality and could be used for identification of high-risk individuals.

## Supporting Information

S1 Table Means (± SD) and prevalence of CVD risk among men, according to the presence of first-degree relatives with CVD and age group. Abbreviations: BMI—body mass index, BP—blood pressure, CVD—cardiovascular disease (myocardial infarction or/and stroke or/and sudden death), SD—standard deviation. *p<0.05, **p<0.01, as compared to the group without first-degree relatives with CVD.
(7Z) Click here for additional data file.
